# Identification of relevant non-target organisms exposed to weevil-resistant Bt sweetpotato in Uganda

**DOI:** 10.1007/s13205-013-0153-1

**Published:** 2013-07-23

**Authors:** R. J. Rukarwa, S. B. Mukasa, B. Odongo, G. Ssemakula, M. Ghislain

**Affiliations:** 1 School of Agricultural Sciences, Makerere University, P.O. Box 7062, Kampala, Uganda; 2 African Institute for Capacity Development, P.O. Box 46179, Nairobi GPO, 00100 Kenya; 3 National Crop Resources Research Institute (NaCRRI), P.O. Box 7084, Namulonge, Kampala, Uganda; 4 International Potato Center, P.O. Box 25171, Nairobi, 00603 Kenya

**Keywords:** Environmental risk assessment, Cry proteins, Sweetpotato weevil

## Abstract

Assessment of the impact of transgenic crops on non-target organisms (NTO) is a prerequisite to their release into the target environment for commercial use. Transgenic sweetpotato varieties expressing Cry proteins (Bt sweetpotato) are under development to provide effective protection against sweetpotato weevils (Coleoptera) which cause severe economic losses in sub-Saharan Africa. Like any other pest control technologies, genetically engineered crops expressing insecticidal proteins need to be evaluated to assess potential negative effects on non-target organisms that provide important services to the ecosystem. Beneficial arthropods in sweetpotato production systems can include pollinators, decomposers, and predators and parasitoids of the target insect pest(s). Non-target arthropod species commonly found in sweetpotato fields that are related taxonomically to the target pests were identified through expert consultation and literature review in Uganda where Bt sweetpotato is expected to be initially evaluated. Results indicate the presence of few relevant non-target Coleopterans that could be affected by Coleopteran Bt sweetpotato varieties: ground, rove and ladybird beetles. These insects are important predators in sweetpotato fields. Additionally, honeybee (hymenoptera) is the main pollinator of sweetpotato and used for honey production. Numerous studies have shown that honeybees are unaffected by the Cry proteins currently deployed which are homologous to those of the weevil-resistant Bt sweetpotato. However, because of their feeding behaviour, Bt sweetpotato represents an extremely low hazard due to negligible exposure. Hence, we conclude that there is good evidence from literature and expert opinion that relevant NTOs in sweetpotato fields are unlikely to be affected by the introduction of Bt sweetpotato in Uganda.

## Weevil-resistant (Bt) sweetpotato for Uganda

Sweetpotato (*Ipomoea batatas* (L) Lam.) is an important crop in all tropical areas of the world. In Uganda, sweetpotato is grown as a staple food in low-input farming systems (Smit 1997). For some farmers, the crop also supplements family income. Strategies to reduce losses due to pests would impact directly on livelihood of millions of rural households by enhancing food security. Sweetpotato weevils, *Cylas puncticollis* Boheman and *C. brunneus* F., are the major production constraints in sub-Saharan Africa (SSA), whereas in the Americas and Asia, *C. formicarius* F. is the major pest (Sorensen 2009). In areas where weevils are endemic, production losses range between 60 and 100 % (Smit 1997; Stathers et al. 2003). In Uganda, a survey on the socioeconomic impact of sweetpotato weevils indicates an average yield loss of over 28 % between wet and dry seasons (B. Kiiza, pers. comm, Makerere University, Kampala, Uganda). Even low levels of *Cylas* spp. infestation can reduce root quality and marketable yield because the plants produce unpalatable terpenoids in response to weevil feeding. In addition, fungal rotting occurring as a consequence of weevil tunnelling in the storage roots produces several compounds including ipomeamarone which is particularly toxic to animals (Pandey 2008). Hence, control of weevils through host plant resistance would bring significant benefits to low-input farmers (Qaim 2001).

Considerable research has been conducted to identify host plant resistance to *Cylas* spp. in sweetpotato and significant progress has been made to release varieties less affected by weevils (Stathers et al. 2003; Jackson et al. 2012; Muyinza et al. 2012). However, improved varieties with high levels of *Cylas* spp. resistance are not yet available. Progress in breeding weevil-resistant cultivars has been slow due to the genetic complexity of the crop (hexaploid and highly heterozygous) and lack of attractiveness of deep-rooting varieties which is the most effective breeding target to avoid weevil infestation (Stathers et al. 2003). Another option could be to breed for enhanced accumulation of the biochemical component of resistance of the variety New Kawogo, but its inheritance and impact on nutritional quality of the storage roots remain to be elucidated (Stevenson et al. 2009). Conversely, genetic transformation for insect resistance is one of the attractive options to improve sweetpotato production as has been witnessed in insect-resistant (Bt) varieties of maize and cotton in sub-Saharan Africa (Thomson 2008). High levels of resistance have been achieved against coleopteran pests by expressing toxins derived from *Bacillus thuringiensis* in the crop plant (Betz et al. 2000; Qaim et al. 2008).

Accordingly, in Uganda Cry proteins were tested for activity against the African sweetpotato weevil resulting in the identification of three samples of *Bacillus thuringiensis* (Bt) endotoxins, Cry7Aa1, ET33/34 and Cry3Ca1 which were found to be active against *C. puncticollis* and *C. brunneus* in artificial diet assays (Ekobu et al. 2010). Therefore, Bt sweetpotato varieties expressing these Cry proteins might be protected against weevils. To that end, the corresponding genes were introduced into sweetpotato via *Agrobacterium tumefaciens* genetic transformation (Ghislain et al. 2013). Assuming weevil pests will be controlled through the expression of the Cry proteins in sweetpotato, it is important to assess the impact of these proteins on other organisms in the sweetpotato growing environments, like other insect control technologies.

## Framework for non-target organisms’ risk assessment of Bt crops

Environmental risk assessment of a Bt crop considers the impact of the Cry protein on the target pest, but also on non-target organisms either directly or indirectly (OECD 2007). To identify relevant non-target organisms, it is important to understand the mode of action of Cry proteins. All Cry toxins characterised so far bind to specific receptors on the plasma membrane of midgut epithelium cells in susceptible insects which form oligomeric transmembrane pores causing osmotic lysis (Aronson and Shai 2001; Bravo et al. 2007). Some Cry proteins have multiple receptors, or may bind to multiple sites on a single receptor and it has been demonstrated that receptor binding is necessary but not sufficient for toxicity (de Maagd et al. 2001). Experiments using sub-lethal concentrations have also revealed that there may be other relevant interactions between Cry proteins and their target insects (Aronson and Shai 2001). Zhang et al. (2006) also suggested that toxicity could be related to G-protein-mediated apoptosis following receptor binding instead of forming oligomers resulting in pore formation. Preliminary research results indicate that the Cry proteins used to control weevils in sweetpotato have similar mode of action as the other Cry proteins (Hernandez-Martinez et al. 2010; B. Escriche, University of Valencia, Spain, pers. comm.).

Non-target organisms (NTO) are species not targeted for control using a particular Cry protein expressed in transgenic plants, but may become exposed to it by feeding directly on plant tissues or indirectly on herbivores or parasites, or by direct ingestion via the environment, such as in the soil or water (Groot and Dicke 2002). Although all organisms of relevance to sweetpotato are arthropods, we will use NTO when discussing non-target arthropods due to the global acceptance of NTO. Non-target risk assessment is a process based on scientific principles that aims at the evaluation of the potential adverse effects of transgenic plants on the non-target organisms of environmental relevance (OECD 2007; Romeis et al. 2008).

## Problem formulation

The initial step of risk assessment is problem formulation which is an important step that leads the risk assessment process to successful risk characterisation (Raybould et al. 2007; Romeis et al. 2011). The Environmental Protection Agency (EPA) of the USA has elaborated in 1998 guidelines on ecological risk assessment which sets the basis for NTO risk assessment (EPA 1998). Problem formulation, in an ideal sense, develops a concise problem statement, a risk hypothesis, a conceptual model and an analysis plan. The risk hypothesis represents an assumption regarding the cause–effect relationships among attributes of the risk characterisation, including sources, exposure routes, end points, responses and measures relevant to the risk assessment. The conceptual model describes key relationships between a transgenic plant occurring in the environment and its linkages to an assessment end point (Raybould 2007). It sets the problem in perspective and establishes the proposed relationships that need evaluation, and the analysis plan establishes the appropriate risk formulation to be considered in the risk characterisation.

## Assessment end points

A second conceptual element of the NTO risk assessment is the assessment end point which is an explicit expression of the environmental value to be protected (EPA 1998). This necessitates defining species and ecosystem functions that could be adversely affected by the Bt plant and that require protection from harm. Assessment end points are made operational into quantitatively measurable end points. An appropriate measurement end point for NTO testing is relative fitness or some component of relative fitness, which is the relative lifetime survival and reproduction of the exposed versus unexposed non-target species (Andow and Hilbeck 2004). It is therefore required that NTO tests consider both toxic effects (mortality, longevity) and sub-lethal effects. The sub-lethal effects are assessed through growth pattern, development rate, reproduction parameters (number and size of offspring, percentage of eggs hatching, sex ratio of progeny, age of sexual maturity), and, when appropriate, behavioural characteristics (searching efficiency, predation rates, food choice). In field conditions, the abundance and species diversity of certain groups of NTO at a relevant life stage are typical measurement end points. The choice of specific measurement end points shall be done according to the problem formulation on a case-by-case basis (Romeis et al. 2011).

## Species selection

Non-target organisms in Bt crop fields feeding directly or indirectly on the crop or residues are exposed to the Cry protein expressed in the pest-resistant plant. Hence, NTO risk assessment has to be done for some of these species when there is a reasonable doubt that they may suffer a negative impact due to a real exposure. For practical reasons, only a small fraction of all possible terrestrial organisms can be considered for regulatory testing. Therefore, to assess the effect of insect-resistant plants on NTO, appropriate species should be selected (Romeis et al. 2008; Garcia-Alonso et al. 2006). It is necessary to select suitable species which can act as surrogates for species that should, but cannot, be tested (Garcia-Alonso et al. 2006; Romeis et al. 2013). The use of appropriate surrogates is a widely accepted concept for scientific experimentation and enables one to design high-quality and repeatable laboratory (and semi-field) studies with clear measurement end points and the ability to extrapolate results to other species. Non-target species subject to the NTO risk assessment should be chosen from different ecological functions such as herbivores, pollinators, predators and parasitoids of pest organisms and decomposers in the soil (Romeis et al. 2006). The NTO risk assessment may also consider species with special aesthetic, economic or cultural value or species of national importance. These species are regionally specific and can be evaluated within the ecological risk assessment independent of their ecological function. To reflect biogeographical variation, it is crucial to determine what relevant species are likely to occur in the cropping systems where the transgenic plant is expected to be grown.

## Framework for NTO risk assessment of Bt sweetpotato in Uganda

In this section, we will apply the NTO risk assessment described above to identify relevant NTOs which could be affected by the cultivation of Bt sweetpotato and recommendations.

## Problem formulation

In Uganda, one possible concern is that Bt sweetpotato plants may have an adverse effect on biodiversity and its functioning at several levels, through interactions with populations of other species associated with Bt sweetpotato fields. Because the environment is to be protected from harm according to protection goals set out by Ugandan legislation (The National Environment Act-Cap 153 1995), protection of species richness and ecological functions should be considered in this risk assessment. The receiving environment is the Bt sweetpotato cultivated fields and the wider environment (other adjacent Bt or non-Bt cultivations). For the benefit of sustainable production, the interest is to maintain a certain level of biodiversity in sweetpotato fields, providing essential functions such as biological control of pests, decomposition of plant materials and maintenance of soil quality and fertility. Bt sweetpotato varieties need to be evaluated to determine if they are directly and/or indirectly (through food web interactions) potentially harmful to species guilds involved in ecosystem functions. Problem formulation in our case is the identification of potential hazards such as exposure to the Cry proteins through a comparison of the Bt sweetpotato with their conventional counterpart.

## Assessment end points

Before commercialisation of Bt sweetpotato, an appropriate assessment end point for initial testing in Bt sweetpotato will be the relative survival and reproduction of NTOs. These parameters are a particularly useful measurable assessment end point in relation to Bt sweetpotato, because they relate directly to risk. Survival experiments should last at least through all relevant developmental stages of the selected NTO, including adult parameters such as age-specific mortality. In principle, the duration of the test should correspond to the time the non-target species would be exposed to the Bt sweetpotato plants or crop residues. In our case, NTO survival experiments would be conducted through all developmental stages, including adult life stage parameters such as age-specific mortality. If the Bt sweetpotato has a negative impact on NTOs in the field, its effect could be observed at any developmental stage during their life cycle. Usually, inappropriate assessment end points may misdirect research or regulatory efforts, and may even lead to the imposition of unnecessary controls to reduce risk.

## Species selection

In tropical ecosystems, there is usually a relatively high number of NTO species that may be exposed to Bt crop plants. Considering that not all species can be evaluated, a representative subset of NTO species should be selected for consideration in the risk assessment based on their known ecological functions. A decision on which focal NTO species are to be used is based on the identification of arthropods associated with the crop and then followed by selection of focal test species.

### Identification of functional groups of arthropods associated with sweetpotato fields

In Uganda like in the rest of Africa, sweetpotato is not a native species, but has been grown long enough to be considered as a traditional food crop. It is a crop grown typically with very little input: sometimes fertilisers but no insecticides or nematicides in SSA. Over the years, field experiments have been conducted in Uganda by National Agricultural Research Organisation (NARO) scientists in different sweetpotato-growing districts to determine pests and beneficial organisms associated with sweetpotato (Ames et al. 1996; Smit 1997; Stathers et al. 2005; Sorensen 2009). Insects representing more than 30 species of eight orders and in different developmental stages were found to be prevalent in sweetpotato fields (Table 1). Their levels of abundance differ according to the seasons and agro-ecological zones. Individuals belonging to eight species of six families were noted as major pests of sweetpotato and are those that farmers have to monitor as part of a sustainable integrated pest management (IPM) system in sub-Saharan Africa (Stathers et al. 2005).

**Table 1:** Insects associated with sweetpotato fields in Uganda (based on insect collection and rearing facility at NaCRRI and literature review)

Order	Family	Species	Common name	Importance	Abundance
Coleoptera	Brentidae	*Cylas puncticolis*	African Sweetpotato weevil	Major Pest	Common
		*Cylas brunneus*	African Sweetpotato weevil	Major Pest	Common
	Scarabeidae	*Phyllophaga* spp.	White grub	Pest	Common
	Meloidae	*Epicauta* spp.	Blister beetle	Pest	Common during flowering
	Curculionidae	*Peloropus batatae*	Peloropus Weevil	Pest	Fairly common
		*Blosyrus obliguatus*	Rough Sweetpotato weevil	Major Pest	Fairly common
		*Alcidodes dentipes*	Striped Sweetpotato weevil	Pest	Fairly Common
	Coccinellidae	*Delphastus catalinae*	Ladybird beetle	Predator	Common
	Chrysomelidae	*Aspidomorpha* spp.	Tortoise shell beetle	Major Pest	Common
	Elateridae	*Hapatesus* spp.	Wireworm	Pest	Common
	Carabidae	*Poecilus chalcites*	Ground beetle	Predator	Common
	Staphylinidae	*Aleochara bilineata*	Rove beetle	Predator	Common
Lepidoptera	Nymphalidae	*Acraea acerata*	Sweetpotato butterfly	Major Pest	Common
		*Synanthedon dascyeles*	Clearwing Moth	Major Pest	Common
	Crambidae	*Leucinodes orbonalis*	Eggplant fruit borer	Pest	Rare
	Noctuidae	*Agrotis subterranea*	Granulate cutworm	Pest	Fairly common
		*Spodoptera* spp.	Armyworm	Major Pest	Common
	Sphingidae	*Agrius cingulata*	Sweetpotato hornworm	Major Pest	Fairly Common
		*Agrius convolvuli*	Sweetpotato moth	Pest	Fairly Common
		*Hippotion celerio*	Taro hawkmoth	Pest	Fairly Common
Isoptera	Termitidae	*Macrotermes bellicosus*	Termite	Pest	Common
Orthoptera	Gryllidae	*Gryllus* spp.	Field cricket	Pest	Common
	Pyrgomoriphidae	*Zonocerous variegatus*	Elegant grasshopper	Pest	Common
	Acrididae	*Attractomorpha psitticina*	Slant-faced grasshopper	Pest	Rare
		*Locusta migratoria*	Migratory locust	Pest	Rare
Hemiptera	Aleyrodidae	*Bemisia tabaci*	Sweetpotato whitefly	Pest/vector	Common
	Aphididae	*Myzus persicae*	Aphid	Pest/vector	Fairly common
		*Macrosiphum euphorbiae*	Potato aphid	Pest	Common
	Coreidae	*Leptoglossus gonagra*	Squash bug	Pest	Common
	Pentatomidae	*Nezara viridula*	Green stink bug	Pest	Common
	Reduviidae	Sycanus spp.	Assassin bug	Predator	Common
Dermaptera	Forficuliidae	*Doru taeniatum*	Earwig	Predator	Common
Hymenoptera	Ichneumonidae	*Charops* spp.	Ichneumon wasp	Parasitoid	Common
	Braconidae	*Meteorus autographae*	Braconid wasp	Parasitoid	Common
	Apidae	*Apis mellifera*	Honeybee	Pollinator	Common
Diptera	Tachinidae	*Caricelia normula*	Tachinid fly	Parasitoid	Common

Furthermore, individuals of 19 species were minor pests, while 9 species belonging to nine families were represented by beneficial insects. The beneficial insects noted were pollinators, decomposers, predators and/or parasitoids of insect pests. Among the homopterans pests observed, white fly (*Bemisia tabaci*) and aphids (e.g. *Myzus persicae*) are vectors of viral diseases. Most of the minor pests observed are cosmopolitan, polyphagous and are pests of other crops. These include *Phyllophaga* spp., *Hapatesus* spp., *Leucinodes orbonalis*, *Spodoptera* spp., *Omopyge sudanica*, *Macrotermes bellicosus*, *Gryllus* spp., *Zonocerous variegates*, *Attractomorpha psitticina*, *Locusta migratoria*, *Bemisiatabaci*, *Myzus persicae*, *Macrosiphum euphorbiae*, *Leptoglossus gonagra* and *Nezara viridula*. In addition, nine spp. of non-insect organisms (Table 2) were also found to be common in sweetpotato fields. Three of these non-insects were beneficial, while five are pests of sweetpotato. The identification of the arthropod complex in sweetpotato fields helps to ascertain value or risk of each species. Samples of arthropod specimen mentioned in this paper have been preserved in the entomology laboratory at National Crop Resources Research Institute (NaCRRI), Namulonge.

**Table 2:** Non-insect arthropods associated with sweetpotato fields in Uganda

Order	Family	Species	Common name	Importance	Abundance
Haplotaxida	Lumbricidae	*Eisenia foetida*	Earthworm	Decomposer	Common
Araneae	Oxyopidae	*Oxyopes* spp.	Lynx spider	Predator	Common
	Glycosidase	*Lycos* Spp.	Wolf spider	Predator	Common
Nematoda	Hoplolaimidae	*Rotylenchulus reniformis*	Reniform nematode	Pest	Common
	Meloidogynidae	*Meloidogyne arenaria*	Root knot nematode	Pest	Common
Rodentia	Muridae	*Mus musculus*	Mouse	Pest	Common
		*Spalax* spp.	Rat	Pest	Common
Diplopoda	Odontopygidae	*Omopyge sudanica*	Millipede	Pest	Common

### Selection of focal species

Based on the considerations addressed on the identification of the functional groups of arthropods associated with sweetpotato fields and categorisation of NTO, focal species need to be selected from each functional category of NTO group. The functional groups commonly associated with sweetpotato fields are pollinators, decomposers, predators and parasitoids. The following criteria should be considered in choosing the appropriate focal test species.

- *The mode of action and specificity of the insecticidal protein and the impact of that protein on non-target species closely related to the target pest* Cry proteins identified as active against *Cylas* spp. (Cry7Aa1, ET33/34, and Cry3Ca1) are typically of the type affecting primarily coleopteran species (Crickmore et al. 2013). Hence, the most likely affected NTO to initially evaluate should be within coleoptera. Therefore, we may consider ground beetles, (Carabidae: *Poecilus chalcites*), rove beetles (Staphylinidae: *Aleochara bilineata*) and ladybird beetles (Coccinellidae: *Delphastus catalinae*) that occur in the same taxonomic order (coleoptera) as the target species. Weevil species such as striped sweetpotato weevil (*Alcidodes dentipes*), rough sweetpotato weevil (*Blosyrus obliguatus*) and peloropus weevil (*Peloropus batatae*) belonging to the same superfamily (Curculionoidea, as the target pests) are either considered as minor pests or not ecologically relevant. There are few examples of cross-order activity for Cry proteins (Tailor et al. 1992; van Frankenhuyzen 2009). However, previous research has shown that that this cross-order activity does not threaten the environmental safety of Bt-based pest control, because Cry proteins tend to be much less toxic to taxa outside of the primary specificity range (van Frankenhuyzen 2009). Furthermore, the large body of published literature provides no indication that the currently grown Bt crops cause direct adverse effects on arthropods that are not closely taxonomically related to the target pest (Romeis et al. 2006; Wolfenbarger et al. 2008; Duan et al. 2010).
- *Exposure based on habitat and field abundance* Relevant NTO should represent species that are abundant in the crop and have known relevant routes of exposure to the insecticidal protein (Romeis et al. 2013). Exposure could be direct, from deliberate or incidental feeding on crop tissues or decaying plant material, or indirect, from feeding on herbivores that feed on the crop. For example, testing ground beetles (Carabidae) is relevant for coleopteran insecticidal proteins produced in sweetpotato, but their exposure is low since these insects are primarily predators of organisms unaffected by Cry proteins in sweetpotato fields, living especially at the soil surface or within the soil where the roots are located. The same can be said of the rove beetle (Staphynilidae). Ladybird beetle (Coccinellidae) adults are active fliers and feed on pollen; they are unlikely to be affected because Bt protein expression in the pollen is low and nectar is a plant secretion, not a tissue and has no cellular content (Ferry et al. 2007). The ladybird larvae feed primarily on aphids feeding on sweetpotato leaves and exposure of the larvae to the Bt toxins is considered to be relatively low, as aphids contain no or only trace amounts of the toxins due to the fact that they feed on the phloem sap which does not contain Cry proteins (Raps et al. 2001; Romeis and Meissle 2011). Although no NTO risk assessment for Bt crops have been conducted on *Delphastus catalinae*, other Coccinellidae species have been shown to be unaffected by coleopteran pest-resistant Bt crops (Duan et al. 2002; Ferry et al. 2007; Li and Romeis 2010; Álvarez-Alfageme et al. 2011). In the case of maize, Cry3Bb1 and Cry34Ab1/Cry35Ab1 proteins’ impact on ground, rove and ladybird species was reviewed and essentially found not to persist in the environment due to rapid degradation in the soil (Wolt et al. 2007). These Cry proteins are closely related to those currently used to engineer resistance to sweetpotato weevils (Cry7Aa1 and Cry3Ca1 are close to the Cry3Bb1 and the ET33/ET34 to the Cry34Ab1/Cry35Ab1) (Crickmore et al. 2013). Furthermore, these Cry proteins used to control weevils in sweetpotato have similar mode of action as the other Cry proteins (B. Escriche, University of Valencia, Spain, pers. comm.). Therefore, Bt sweetpotato is unlikely to cause harm to the above-mentioned species.
- *Ecological and taxonomic diversity* Relevant NTO may include a broad range of invertebrates, particularly economically or socially beneficial species that represent diverse habitats. In our case; honeybee (*Apis mellifera)* is the main pollinator of sweetpotato, which may forage for sweetpotato pollen and therefore could be exposed to Cry proteins. Earthworms (*Eisenia foetida*) are important decomposers, and sweetpotato butterfly (*Acraea acerata*) feeds on plant canopy and would be surrogate to lepidopteron arthropods which feed on the sweetpotato canopy. In all three cases, coleopteran-specific Cry proteins are unlikely to cause any harm because of their target specificity. Indeed, a meta-analysis of Cry protein impact on honeybees resulted in no harm, as shown by recent studies (Duan et al. 2008; Hendriksma et al. 2012). Similarly, field studies have also shown no significant differences in earthworm populations in fields planted with Cry1Ab1 or Cry3Bb1 proteins (van der Merwe et al. 2012).
- *Ability to conservatively estimate field exposure* In the laboratory, the potential level of exposure of test species to insecticidal proteins in the field has to be identified. Farmers rarely cultivate only one landrace in one area; even when they have adopted an improved variety, they will maintain some level of diversity because production does not target a single use. It is unlikely that a Bt sweetpotato improved variety will displace landraces, because these are grown for their culinary or taste properties. Therefore, an accurate estimate of exposure of the relevant NTO will be difficult to agree on. Hence, the concentration of the insecticidal protein in the plant tissue on which the NTO feeds provides a worst-case estimate of the environmental exposure concentration. Such data for the Bt sweetpotato is not yet available, because Bt sweetpotato is still under development in Uganda.
- *Whether a suitable test system exists for laboratory analysis* Relevant NTOs adaptable to a laboratory bioassay system and suitable protocols are necessary for testing. When feasible, the organism life stage that is most susceptible to the insecticidal protein should be tested. Protocols typically include information on test end points, positive/negative controls, acceptable control mortality, sample sizes and statistical power analyses. For a number of chosen species on Bt sweetpotato, standard testing protocols are not yet available but a number of protocols are available from tests conducted with other crops and/or related invertebrate species, which could be adapted for testing the effect of insecticidal proteins being expressed in sweetpotato.

In general, non-target organisms that are related taxonomically to the target pests are most likely to be affected similarly by the Bt Cry protein (Romeis et al. 2008). In the case of Bt sweetpotato, the rove, ground and ladybird beetles are the primary relevant NTO. Accepting a much lower probability of impact, the honeybee as the main pollinator of sweetpotato and a charismatic insect may be looked at as an NTO for Bt sweetpotato. However, numerous impact studies have been published over the last decade and have been subject to meta-analyses drawing very clear conclusions of non-impact of Cry proteins on NTO (Marvier et al. 2007; Wolt et al. 2007; Duan et al. 2008; Wolfenbarger et al. 2008). Species that are not exposed to the Cry proteins or from kingdoms never reported to be affected by other Cry proteins do not need to be tested to draw a negligible-risk conclusion (Peterson et al. 2011; Prischl et al. 2012).

**Fig. 1:**
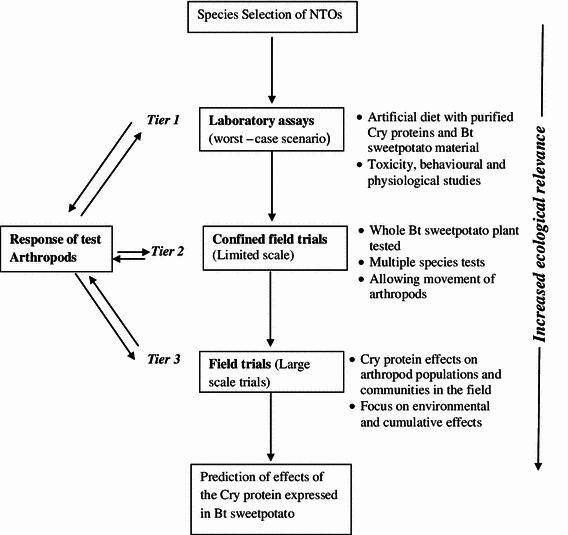
Tiered approach for testing the effect of Cry proteins non-target organisms found in Bt sweetpotato fields

Once the relevant non-target species are selected and their surrogates identified, these would be evaluated moving through the tiered testing procedure that has been recommended and well accepted by regulators and risk assessors (Garcia-Alonso et al. 2006; Romeis et al. 2006, 2008; USEPA 2007). In the case of ground, rove and ladybird beetles, these can be used directly as test species. The procedure starts with laboratory tests (lower tier), followed by semi-field (glasshouse or screenhouse) and field (higher tier) tests if necessary (Fig. 1). However, the tiers should not be just considered as sequential steps in a linear approach, because the response of arthropods between the tiers is necessary during the assessment, to determine whether to stop or proceed to the next tier (Kos et al. 2009). Lower-tier tests serve to identify potential hazards and are typically conducted in controlled conditions. Lower-tier tests are designed to measure a specific end point under worst-case conditions using protein concentrations that are normally 10–100 times higher than those present in plant tissues (USEPA 2007). Lower-tier studies must be properly designed and executed to maximise the probability that compounds with adverse effects are detected. The confidence in the conclusions drawn from these studies mainly depends on the study’s ability to detect potential hazards, if present (Romeis et al. 2008; Duan et al. 2010). The Cry protein level of the Cry7Aa1, ET33/ET34 and Cry3Ca1 will first be determined for the transgenic events causing mortality in both the storage roots and leaves, and then 10–100 times higher than those present in these tissues will be evaluated in artificial diet bioassays. The use of storage root-specific promoters (sporamin and β-amylase) in sweetpotato is expected to reduce the amount of Cry proteins in leaves.

## Conclusion

This review provides the scientific rationale for risk assessment of Bt sweetpotato to assist regulatory decision making. The risk hypotheses are developed from current knowledge about the crop, the Cry proteins, the receiving environment and the interactions of the three. It therefore makes maximum use of the existing data and aims to minimise collection of data that are irrelevant to the risk assessment of non-target arthropods. Accordingly, we have identified the ground, rove and ladybird beetles as the primary relevant NTOs which may potentially be affected by cultivation of Bt sweetpotato. Honeybees may be considered as relevant due to their ecological role, but there is solid scientific evidence from literature indicating no harm. Potential hazards are evaluated with representative surrogate/indicator species that are selected case by case for their suitability and amenability to test relevant risk. For effective NTO assessment threshold, values need to be defined that elicit the advance to higher tiers as has been done for environmental risk assessments of conventional pesticides. These values will be available when a transgenic event with efficacy to control weevils will be available. At this point, it is too speculative to estimate what this threshold could be based on solely the LC_50_ which is currently the only toxicity value known. Tissue-specific or enhanced promoters and different Cry protein combinations will influence this threshold value. It is important to note that defining the threshold values is not solely a scientific question, but also depends on whether policy makers are concerned about under- or over-estimating risks considering that sweetpotato is an introduced crop and that Bt sweetpotato will bring food security benefits to vulnerable populations. The NTO testing approach presented above minimises the likelihood of unexpected negative impact on other organisms and help decision makers to authorise the release of weevil-resistant sweetpotato plants with confidence that it will not have undesirable effects on NTOs.

## References

[CR1] Álvarez-Alfageme F, Bigler F, Romeis J (2011). Laboratory toxicity studies demonstrate no adverse effects of Cry1Ab and Cry3Bb1 to larvae of *Adalia bipunctata* (*Coleoptera: Coccinellidae*): the importance of study design. Transgenic Res.

[CR2] Ames T, Smit NEJM, Braun AR, O’Sullivan JN, Skoglund L (1996). Sweetpotato: major pests diseases and nutritional disorders.

[CR3] Andow DA, Hilbeck A (2004). Science-based risk assessment for non-target effects of transgenic crops. BioSci.

[CR4] Aronson AI, Shai Y (2001). Why *Bacillus thuringiensis* insecticidal toxins are so effective: unique features of their mode of action. FEMS Microbiol Lett.

[CR5] Betz FS, Hammond BG, Fuchs RL (2000). Safety and advantages of *Bacillus thuringiensis*-protected plants to control insect pests. Regul Toxicol Pharm.

[CR6] Bravo A, Gill SS, Soberón M (2007). Mode of action of *Bacillus thuringiensis* Cry and Cyt toxins and their potential for insect control. Toxicon.

[CR7] Crickmore N, Baum J, Bravo A, Lereclus D, Narva K, Sampson K, Schnepf E, Sun M, Zeigler DR (2013) *Bacillus thuringiensis* toxin nomenclature. http://www.btnomenclature.info/. Accessed 20 Mar 2013

[CR8] de Maagd RA, Bravo A, Crickmore N (2001). How *Bacillus thuringiensis* has evolved specific toxins to colonize the insect world. Trends Genet.

[CR9] Duan JJ, Head G, McKee MJ, Nickson TE, Martin JW, Sayegh FS (2002). Evaluation of dietary effects of transgenic corn pollen expressing Cry3Bb1 protein on a non-target ladybird beetle, *Coleomegilla maculata*. Entomol Exp Appl.

[CR10] Duan JJ, Marvier M, Huesing J, Dively G, Huang ZY (2008). A meta-analysis of effects of Bt crops on honey bees (Hymenoptera: Apidae). PLoS ONE.

[CR11] Duan JJ, Lundgren JG, Naranjo S, Marvier M (2010). Extrapolating non-target risk of Bt crops from laboratory to field. Biol Lett.

[CR12] Ekobu M, Solera M, Kyamanywa S, Mwanga ROM, Odongo B, Ghislain M, Moar WJ (2010). Toxicity of seven Bacillus thuringiensis Cry proteins against *Cylas puncticollis* and *Cylas brunneus* (Coleoptera: Brentidae) using a novel artificial diet. J Econ Entomol.

[CR13] EPA (1998) Guidelines for Ecological Risk Assessment. EPA/630/R-95/002F, April 1998 Final. United States Environmental Protection Agency. Washington, D.C

[CR14] Ferry N, Mulligan EA, Majerus ME, Gatehouse AM (2007). Bitrophic and tritrophic effects of Bt Cry3A transgenic potato on beneficial, non-target, beetles. Transgenic Res.

[CR15] Garcia-Alonso M, Jacobs E, Raybould A, Nickson TE, Sowig P, Willekens H, van der Kouwe P, Layton R, Amijee F, Fuentes AM, Tencalla F (2006). A tiered system for assessing the risk of genetically modified plants to non-target organisms. Environ Biosaf Res.

[CR16] Ghislain M, Tovar J, Prentice K, Ormachea M, Rivera C, Manrique S, Kreuze J, Rukarwa R, Sefasi A, Mukasa S, Ssemakula G, Wamalwa L, Machuka J (2013). Weevil resistant sweetpotato through biotechnology. Acta Hortic (ISHS).

[CR17] Groot AT, Dicke M (2002). Insect-resistant transgenic plants in a multi-trophic context. Plant J.

[CR18] Hendriksma HP, Härtel S, Babendreier D, von der Ohe W, Steffan-Dewenter I (2012) Effects of multiple Bt proteins and GNA lectin on in vitro-reared honey bee larvae. Apidologie 1–12. doi: 10.1007/s13592-012-0123-3 10.1371/journal.pone.0028174 PMC3241620 22194811

[CR19] Hernandez-Martinez P, Moar W, Escriche B (2010) Proteolytic processing of *Bacillus thuringiensis* Cry7Aa toxin and specific binding to brush border membrane vesicles of three sweetpotato weevil species (Coleoptera: Brentidae), abstract presented at the 43th meeting of the Society for Invertebrate Pathology, 11–15 May Trabzon, Turkey

[CR20] Jackson DM, Harrison HF, Ryan-Bohac JR (2012). Insect resistance in sweetpotato plant introduction accessions. J Econ Entomol.

[CR21] Kos M, van Loon JJ, Dicke M, Vet LE (2009). Transgenic plants as vital components of integrated pest management. Trends Biotechnol.

[CR22] Li Y, Romeis J (2010). *Bt* maize expressing Cry3Bb1 does not harm the spider mite, *Tetranychus urticae*, or its ladybird beetle predator, *Stethorus punctillum*. Biol Control.

[CR23] Marvier M, McCreedy C, Regetz J, Kareiva P (2007). A meta-analysis of effects of Bt cotton and maize on nontarget invertebrates. Science.

[CR24] Muyinza H, Talwana HL, Mwanga RO, Stevenson PC (2012). Sweetpotato weevil (*Cylas* spp.) resistance in African sweetpotato germplasm. Int J Pest Manage.

[CR25] OECD (2007). Consensus document on safety information on transgenic plants expressing *Bacillus thuringiensis*-derived insect control protein. Series on harmonisation of regulatory oversight in biotechnology.

[CR26] Pandey G (2008). Acute toxicity of ipomeamarone, a phytotoxin isolated from the injured sweet potato. Pharmacogn Mag.

[CR27] Peterson JA, Lundgren JG, Harwood JD (2011). Interactions of transgenic *Bacillus thuringiensis* insecticidal crops with spiders (Araneae). J Arachnol.

[CR28] Prischl M, Hackl E, Pastar M, Pfeiffer S, Sessitsch A (2012). Genetically modified Bt maize lines containing *cry3Bb1*, *cry1A105* or *cry1Ab2* do not affect the structure and functioning of root-associated endophyte communities. Appl Soil Ecol.

[CR29] Qaim M (2001). A prospective evaluation of biotechnology in semi-subsistence agriculture. Agr Econ.

[CR30] Qaim M, Pray C, Zilberman D, Romeis J, Shelton A, Kennedy G (2008). Economic and social considerations in the adoption of Bt Crops. Integration of insect-resistant genetically modified crops within IPM programs.

[CR31] Raps A, Kehr J, Gugerli P, Moar WJ, Bigler F, Hilbeck A (2001). Immunological analysis of phloem sap of *Bacillus thuringiensis* corn and of the non-target herbivore *Rhopalosiphum padi* (Homoptera: Aphididae) for the presence of Cry1Ab. Mol Ecol.

[CR32] Raybould A (2007). Environmental risk assessment of genetically modified crops: general principles and risks to non-target organisms. BioAssay.

[CR33] Raybould A, Stacey D, Vlachos D, Graser G, Li X, Joseph R (2007). Non-target organisms risk assessment of MIR604 maize expressing mCry3A for control of corn rootworms. J Appl Entomol.

[CR34] Romeis J, Meissle M (2011). Non-target risk assessment of Bt crops-cry protein uptake by aphids. J Appl Ent.

[CR35] Romeis J, Meissle M, Bigler F (2006). Transgenic crops expressing *Bacillus thuringiensis* toxins and biological control. Nature Biotechnol.

[CR36] Romeis J, Barsch D, Bigler F, Candolfi MP, Gielkens MMC, Hartley SE, Hellmich RI, Huesing JE, Jepson PC, Layton R, Quemada H, Raybould A, Rose RI, Schiemann J, Sears MK, Shelton AM, Sweet J, Vaituzis Z, Wolt JD (2008). Assessment of risk of insect-resistant transgenic crops to non target arthropods. Nature Biotechnol.

[CR37] Romeis J, Hellmich RL, Candolfi MP, Carstens K, De Schrijver A, Gatehouse AMR, Herman RA, Huesing JE, McLean MA, Raybould A, Shelton AM, Waggoner A (2011). Recommendations for the design of laboratory studies on non-target arthropods for risk assessment of genetically engineered plants. Transgenic Res.

[CR38] Romeis J, Raybould A, Bigler F, Candolfi MP, Hellmich RL, Huesing JE, Shelton AM (2013). Deriving criteria to select arthropod species for laboratory tests to assess the ecological risks from cultivating arthropod-resistant genetically engineered crops. Chemosphere.

[CR39] Smit NEJM (1997) Integrated Pest Management for sweetpotato in Eastern Africa. PhD Thesis, Agricultural University Wageningen, The Netherlands

[CR40] Sorensen KA, Loebenstein G, Thottappilly G (2009). Sweetpotato insects: identification, biology and management. The Sweetpotato.

[CR41] Stathers TE, Rees D, Kabi S, Mbilinyi L, Smit NEJM, Kiozya H, Jeremiah S, Nyango A, Jeffries D (2003). Sweetpotato infestation by *Cylas* spp. in East Africa: I: Cultivar differences in field infestation and the role of plant factors. Int J Pest Manage.

[CR42] Stathers T, Namanda S, Mwanga ROM, Khisa G, Kapinga R (2005). Manual for sweetpotato integrated production and pest management farmer field schools in sub-Saharan Africa.

[CR43] Stevenson PC, Muyinza H, Hall DR, Porter EA, Farman D, Talwana H, Mwanga ROM (2009). Chemical basis for resistance in sweetpotato *Ipomoea batatas* to the sweetpotato weevil *Cylas puncticollis*. Pure Appl Chem.

[CR44] Tailor R, Teppett J, Gibb G, Stephen P, Derek P, Linda J, Ely S (1992). Identification and characterization of a novel *Bacillus thuringiensis* δ -endotoxin entomocidal to coleopteran and lepidopteran larvae. Mol Microbiol.

[CR45] Thomson JA (2008). The role of biotechnology for agricultural sustainability in Africa. Philos Trans R Soc Lond B Biol Sci.

[CR46] USEPA (2007) White paper on tier-based testing for the effects of proteinaceous insecticidal plant-incorporated protectants on non-target arthropods for regulatory risk assessments. U.S. Environmental Protection Agency (USEPA), Washington D. C. http://www.epa.gov/pesticides/biopesti-cides/pips/non-target-arthropods.pdf. Accessed 20 Jan 2013

[CR47] van der Merwe F, Bezuidenhout C, van den Berg J, Maboeta M (2012). Effects of Cry1Ab transgenic maize on lifecycle and biomarker responses of the earthworm, *Eisenia Andrei*. Sensors.

[CR48] van Frankenhuyzen K (2009). Insecticidal activity of *Bacillus thuringiensis* crystal proteins. J Invertebr Pathol.

[CR49] Wolfenbarger LL, Naranjo SE, Lundgren JG, Bitzer RJ, Watrud LS (2008). Bt crops effects on functional guilds of non-target arthropods: a meta-analysis. PLoS ONE.

[CR50] Wolt JD, Prasifka JR, Hellmich RL (2007) Ecological safety assessment of insecticidal proteins introduced into biotech crops. In: Hammond BG (ed) Food safety of proteins in agricultural biotechnology, vol 172. CRC Press. doi:10.1201/9781420005738.ch4

[CR51] Zhang X, Candas M, Griko NB, Taussig R, Bulla LA (2006). A mechanism of cell death involving an adenylyl cyclase/PKA signaling pathway is induced by the Cry1Ab toxin of *Bacillus thuringiensis*. Proc Natl Acad Sci.

